# Central Precocious Puberty – Management and Long-term Outcomes

**DOI:** 10.17925/EE.2015.11.01.45

**Published:** 2015-04-11

**Authors:** Juliane Léger, Jean-Claude Carel

**Affiliations:** 1. Assistance Publique-Hôpitaux de Paris, Hôpital Robert Debré, Service d’Endocrinoiogie Diabetologie Pédiatrique, Centre de Référence des Maladies Endocriniennes Rares de la Croissance, F-75019 Paris, France; 2. Université Paris Diderot, Sorbonne Paris Cité, F-75019 Paris, France; 3. Institut National de la Santé et de la Recherche Médicale (Inserm), Unité 1141, DHU Protect, F-75019 Paris, France

**Keywords:** Precocious puberty, treatment, outcome

## Abstract

Central precocious puberty (CPP) results from premature re-activation of the gonadotropic axis. CPP is much more common in girls than in boys and is idiopathic in most cases. In boys, precocious puberty is more likely to be linked to hypothalamic lesions (≈40%). Recent studies have implicated the inactivation of *MKRN3* gene in ‘idiopathic’ CPP Gonadotropin-releasing hormone agonists are the standard treatment for progressive CPP.

Precocious puberty (PP) is defined as the onset of clinical signs of puberty before age 8 years in girls and 9.5 years in boys. However, the onset of puberty may be subject to constitutional (genetics, ethnicity) environmental (secular trends, adoption, absence of the father and possible exposure to oestrogenic endocrine-disrupting chemicals) and nutritional (body mass index) variations,^1–3^ with implications for the definition of precocious puberty. The signs of puberty include breast development in girls and testicular enlargement in boys (testicular volume greater than 4 ml or testicular length greater than 25 mm).

PP leads to the progressive development of secondary sexual characteristics, together with the development of pubic hair, and an acceleration of growth velocity and bone maturation, resulting in premature fusion of the growth plates, potentially responsible for adult height deficit. It may have consequences for growth and psychosocial development. PP may be caused by central or peripheral mechanisms.^1^

Central PP (CPP), which is much more common in girls than in boys,^4^ results from premature reactivation of the hypothalamo-pituitary-gonadal axis and pulsatile gonadotropin-releasing hormone (GnRH) secretion, with a hormonal pattern similar to that of normal puberty. CPP may be due to hypothalamic lesions but is idiopathic in most cases, particularly in girls^1^. Recent studies have implicated the activation of Kisspeptin and its receptor and the inactivation of Makorin ring finger 3 *(MKRN3)* genes in “idiopathic” CPP^5–6^. *MKRN3* is an imprinted gene located on the long arm of chromosome 15, with a potentially inhibitory effect on GnRH secretion. *MKRN3* gene defects have been identified as a cause of paternally transmitted familial CPP, but such defects do not underlie maternally transmitted CPP and are rarely involved in sporadic forms.

Premature sexual maturation is a frequent cause for referral. Clinical evaluation is generally sufficient to reassure the patients and their families, but premature sexual maturation may reveal severe conditions, and thorough evaluation is therefore required to identify its cause and potential for progression so that appropriate treatment can be proposed. If a non-progressive form of PP is suspected, it is recommended to wait a few months and then to reassess the patient, to avoid unnecessary treatment. The heterogeneity of CPP, in terms of its clinical presentation and definition, can be accounted for by the gradual nature of the transition to puberty. Indeed, in many girls with idiopathic CPP, puberty progresses very slowly and may even be regressive, resulting in an unchanged predicted final stature and a normal adult height close to parental target height.^7,8^ Therapeutic abstention is appropriate in most cases, because puberty progresses slowly, with the menarche occurring, on average, 5.5 years after the onset of clinical signs of puberty and normal adult height relative to parental target height being reached. However, in some cases (about one-third of subjects), final stature prognosis may worsen during the progression of puberty, in parallel with the emergence of evident biological signs of oestrogenisation. Clinical assessments should therefore be systematically carried out in children for whom no treatment is justified at the initial assessment, at least until the age of 9 years, to identify girls subsequently requiring treatment to block precocious puberty.^1,8,9^

In both sexes, the central cause of precocious puberty is demonstrated by an increase in pituitary gonadotropin levels. Indeed, the mechanism of precocious puberty involves premature activation of the hypothalamic-pituitary-gonadal axis, with the initiation of pulsatile luteinising hormone (LH) secretion and an increase in the secretion of pituitary gonadotropins, both in basal conditions and after stimulation with LH-releasing hormone (LHRH). Before the onset of puberty, the follicle-stimulating hormone (FSH) peak is greater than the LH surge. During and after puberty, the LH surge predominates. In cases of central precocious puberty, basal serum LH concentration is usually ≥0.3 IU/l and LH concentration after stimulation is ≥5 IU/l.^1,10^ Oestrogenic impregnation is assessed on pelvic ultrasound scans, which may show an oestrogenised appearance of the uterus (length ≥35 mm).^11^ Central magnetic resonance imaging focusing on the hypothalamic region is required in most cases of CPP^1,12^

GnRH agonists (GnRHa) are the standard treatment for progressive CPP.^9,12^ Such treatment results in the regression or stabilisation of pubertal symptoms and decreases in growth velocity and bone age advancement. The factors affecting height outcome include initial patient characteristics and treatment duration. After the cessation of GnRHa therapy, generally at an age of about 11 years, biological and clinical signs of puberty reappear within months, with most girls achieving menarche, with menstrual ovulation cycles, during the following year.^9,13,14^ PP associated with the presence of a hypothalamic lesion may progress to gonadotropin deficiency. The available data indicate that long-term GnRHa treatment does not seem to cause or aggravate obesity or have repercussions for body composition, bone mineral density, fertility and metabolic or cancer comorbidities. General health status is not different from that of women with normal puberty.^14,15^ However, data concerning psychosocial outcomes are scarce,^15,16^ and studies of this aspect are required.
